# Zwischen Akte und Krankenakte: Ein Fallbeispiel aus dem Stasi-Haftkrankenhaus Berlin-Hohenschönhausen

**DOI:** 10.1007/s00048-026-00449-5

**Published:** 2026-07-30

**Authors:** Tilman Wickert, Kathleen Haack, Felicitas Soehner, Ekkehardt Kumbier

**Affiliations:** Arbeitsbereich Geschichte der Medizin, Universitätsmedizin Rostock, Doberaner Str. 140, 18057 Rostock, Deutschland

**Keywords:** DDR, Psychiatrie, Staatssicherheit, Untersuchungshaft, Medikamente, GDR, Psychiatry, State security, Pre-trial detention, Medication

## Abstract

Der gezielte Einsatz von Medikamenten in Untersuchungshaftanstalten des MfS vor Vernehmungen ist bislang nur unzureichend empirisch belegt. Die Akten des Untersuchungshäftlings „Clausmann“ liefern jedoch erstmals Anhaltspunkte dafür, dass psychotrope Substanzen möglicherweise gezielt vor Verhören verabreicht wurden und deren Anwendung gegenüber Gerichtsinstanzen nicht vollständig offengelegt wurde. Vor dem Hintergrund der institutionellen Funktion des Haftkrankenhauses Berlin-Hohenschönhausen innerhalb des Sicherheitsapparates der DDR untersucht der Beitrag exemplarisch, in welchem Ausmaß medikamentöse Maßnahmen in die geheimpolizeilichen Praktiken des Ministeriums für Staatssicherheit eingebunden gewesen sein könnten und welche Bedeutung dies für die historische Bewertung der Untersuchungshaftpraxis sowie für zukünftige Forschungsfragen hat.

## Abstract (en)

The targeted use of medication in Stasi remand detention centers prior to interrogations has so far been insufficiently empirically documented. However, the files of the pre-trial detainee “Clausmann” provide, for the first time, evidence that psychotropic substances may have been deliberately used prior to interrogations and that their use was not fully disclosed to the courts. Against the backdrop of the institutional function of the Berlin-Hohenschönhausen prison hospital within the GDR’s security apparatus, this article examines, by way of example, the extent to which medication may have been integrated into the secret police practices of the Ministry for State Security and its significance for the historical evaluation of pre-trial detention practices and for future research questions.

## Einleitung

Die Frage, ob das Ministerium für Staatssicherheit der DDR im Rahmen von Vernehmungen gezielt psychotrop wirkende Medikamente in ihren Untersuchungshaftanstalten (UHA) einsetzte, wurde seitens ehemaliger Häftlinge wiederholt bejaht (Söhner et al. 2025). Für die zeithistorische Forschung blieb diese Frage bisher offen, da es abgesehen von mündlichen Aussagen weitgehend an schriftlichen Quellen fehlt, die diese Vorwürfe bestätigen oder widerlegen könnten. Das ist auch darauf zurückzuführen, dass die Verschränkung von geheimpolizeilicher Praxis und medizinischer Expertise innerhalb der Strukturen des Ministeriums für Staatssicherheit (MfS) nach wie vor ein Forschungsdesiderat darstellt. Weder der Zentrale Medizinische Dienst (ZMD) des MfS noch das ihm unterstellte MfS-Haftkrankenhaus in Berlin-Hohenschönhausen wurden bisher umfassend erforscht. Beide Einrichtungen bildeten jedoch entscheidende Schnittstellen, an denen medizinische Expertise mit den operativen Praktiken der Geheimpolizei zusammentraf.

Ziel des Beitrags ist es, am Beispiel eines konkreten Falls – im Folgenden fiktiv und pseudonymisiert als „Clausmann“ benannt – zu rekonstruieren, in welchem Umfang die gefängnismedizinische Expertise und die Abläufe im Haftkrankenhaus in die geheimpolizeiliche Praxis der Stasi eingebunden waren. Im Mittelpunkt steht die Frage, ob medizinische Maßnahmen während der Untersuchungshaft dazu dienten, Informationen zu gewinnen und Geständnisse zu erzwingen.

## Erkenntnisstand und Methodik

Trotz wichtiger medizin- und zeithistorischer Forschungen zur Geschichte der DDR blieben das Haftkrankenhaus Hohenschönhausen sowie der Zentrale Medizinische Dienst weitgehend unerforscht. Über den ZMD existiert bislang lediglich ein kurzer Aufsatz von Heidrun Budde (2015). Bisherige Arbeiten – wie die von Süß (1998: 438–462) oder Voigt/Erler (2011) – lieferten erste Erkenntnisse zu Struktur, Personalbestand und grundsätzlicher Organisation. Sie konnten jedoch die Details der geheimpolizeilichen Praxis und die Rolle des medizinischen Personals kaum rekonstruieren. Der zunächst eingeschränkte Zugang zu personenbezogenen Haft- und Krankenakten erschwerte eine eigenständige Analyse erheblich, was Voigt/Erler ausdrücklich betonen (Voigt & Erler 2011: 4–7). Vor diesem Hintergrund beschränkten sich die Forschenden überwiegend auf Kaderakten relevanter MfS-Mitarbeiterinnen und -Mitarbeiter oder auf Dissertationen, die an der Juristischen Hochschule des MfS erstellt wurden. Mit den Novellierungen des Stasiunterlagengesetzes (siebte und achte Novelle) entstanden weitere Forschungen, die über institutionelle Strukturbeschreibungen hinaus auch Funktionen und Örtlichkeiten der MfS-Untersuchungshaft auf Basis von Inhaftiertenakten untersuchten.^1^ Das Haftkrankenhaus und die Rolle des ZMD blieben jedoch größtenteils unberücksichtigt. Eine Ausnahme bildet die Studie von Spohr zur UHA Hohenschönhausen, die dem Haftkrankenhaus einen kurzen Exkurs widmet (Spohr 2015: 227–229). Auch die Forschungen zur „Operativen Psychologie“ des MfS oder zu medizinisch-psychologischen Ansätzen lassen das Haftkrankenhaus und den ZMD weitgehend außen vor (Wieser 2025). Mehr Aufmerksamkeit erhielt das Haftkrankenhaus in neueren Arbeiten, etwa in einem Aufsatz von Erler zu ungeklärten Todesfällen in MfS-Haft (Erler 2015: 65–84). Darüber hinaus liegen mittlerweile vertiefte Forschungen zum direkten institutionellen Umfeld des Haftkrankenhauses vor, auf die Süß und Voigt/Erler noch verzichten mussten. Besonders hervorzuheben ist die Monografie der BStU-Forschungsabteilung zur Hauptabteilung IX, die als Untersuchungsorgan des MfS maßgeblich die Ermittlungen gegen Häftlinge leitete und offenbar Einfluss auf die Arbeit der Ärzte im Haftkrankenhaus ausübte (Engelmann & Joestel 2016).

Studien zur Rolle von Rechtsanwälten und Staatsanwaltschaften in politischen Strafprozessen der DDR haben zudem die Interaktion und das strategische Zusammenwirken von MfS und Gerichtsakteuren detailliert herausgearbeitet (Booß 2017; Booß & Richter 2024). Diese Erkenntnisse sind für die Analyse des Haftkrankenhauses von zentraler Bedeutung, da sie das unmittelbare institutionelle Umfeld beschreiben, mit dem das medizinische Personal interagierte. Zudem sind medizinhistorische Forschungen zur Psychiatrie in der DDR hervorzuheben. Sie zeigen, dass die Psychiatrie in der DDR neben Missbrauch und Verwahrung durchaus auch Hilfe bot.^2^ Für die geheimdienstgeschichtliche und zeithistorische Forschung sind diese Ergebnisse von Bedeutung, da sie einerseits die Grenzen geheimpolizeilicher Eingriffe im Gesundheitswesen aufzeigen, andererseits aber auch deutlich machen, dass menschenrechtsverletzende Missstände nicht immer gezielt durch die Stasi verursacht wurden.

Insgesamt lässt sich festhalten, dass in den letzten zehn Jahren die Forschung zwar nicht direkt zum Haftkrankenhaus und zum ZMD, aber zu deren Umfeld ausgeweitet wurde. In Kombination mit der verbesserten Quellenlage ist eine fundierte Einordnung der medizinischen Praxis innerhalb der operativen Strukturen und geheimpolizeilichen Funktionsmuster des MfS tendenziell möglich.

Ein erster Aufschlag dazu erfolgte unlängst in einer kurzen medizinhistorischen Kasuistik (Wickert et al. 2026). Aufbauend darauf erscheint es notwendig, die Fallanalyse deutlich zu erweitern. Nur so lässt sich die komplexe Verflechtung von medizinischer Praxis, geheimdienstlicher Strategie und individueller Entscheidungsfindung im Haftkrankenhaus Hohenschönhausen besser nachvollziehen. Eine solch detaillierte Fallbeschreibung ermöglicht es, die Abläufe innerhalb der Einrichtung differenziert zu rekonstruieren, die Rolle des medizinischen Personals zu beleuchten und die Schnittstellen zur Hauptabteilung IX des MfS präzise aufzuzeigen. Zudem ermöglicht ein vertiefter Blick in den Einzelfall, die vermuteten medikamentösen Interventionen im Rahmen von Vernehmungen zu untersuchen und deren Funktion innerhalb der geheimdienstlichen Praxis zu bewerten. Durch die Kombination von Haft- und Krankenakten lassen sich erstmals Zusammenhänge zwischen medizinischen Maßnahmen, Vernehmungsstrategien und der institutionellen Logik des MfS rekonstruieren. Auf diese Weise kann eine erste belastbare Grundlage für die Bewertung des Einsatzes gefängnismedizinischer Expertise im Kontext von Macht- und Herrschaftsausübung in der DDR geliefert werden.

Die methodische Basis bildet die Analyse schriftlicher Überlieferungen des MfS im Bundesarchiv. Im Zentrum stehen zum einen die Kommunikation der Linie IX mit Staatsanwaltschaften und Gerichten in Form von Prozess- und Haftakten, die unter anderem prozessrelevante Dokumente wie Vernehmungsprotokolle, Beweismitteldokumentationen und Anklageschriften enthalten; zum anderen die überlieferten Krankenakten des Haftkrankenhauses Berlin-Hohenschönhausen. Durch den Abgleich beider Quellengruppen lässt sich die mögliche Doppelfunktion medizinischer Maßnahmen untersuchen: ob sie ausschließlich der gefängnismedizinischen Versorgung dienten oder zugleich Bestandteil strategischer Praktiken der Staatssicherheit waren – etwa zur Beeinflussung von Untersuchungshäftlingen. Gerade im Hinblick auf die medizinische Dimension eröffnet der kombinierte Quellenzugang – Außenkommunikation und Krankenakten – neue Erkenntnismöglichkeiten. Er erlaubt die Nachzeichnung, ob und wann medizinische Handlungen von der Linie IX als geheimpolizeiliche Maßnahmen behandelt, verschleiert oder aber als reguläre Dokumente einer medizinischen Gesundheitsversorgung an Gerichte weitergeleitet wurden. Im Folgenden soll deshalb die Verschränkung von medizinischen und geheimpolizeilichen Maßnahmen aufgezeigt werden. Im Fokus stehen dabei die Fahndungs- und Kriminalisierungsbemühungen des MfS gegen den Untersuchungshäftling „Clausmann“ sowie dessen medizinische Behandlung im Haftkrankenhaus Berlin-Hohenschönhausen.

## Das Haftkrankenhaus im Gefüge der MfS-Strukturen

Das Haftkrankenhaus in Berlin-Hohenschönhausen war das zentrale Haftkrankenhaus des MfS. Seit 1945 war das Gelände vom sowjetischen Geheimdienst genutzt worden, bevor es 1951 an die DDR überging. Die historische Bedeutung dieses Ortes ist kaum zu überschätzen: Kein anderer Gefängniskomplex der DDR verdeutlicht so klar das Ineinandergreifen von Isolation, psychologischer Beeinflussung und medizinischer Kontrolle. Nach seiner Gründung 1959 fungierte es als spezialisierte und anfangs eigenständige Hafteinrichtung, ehe es 1973 dem ZMD des MfS unterstellt wurde (Erler 2015: 77). Streng geheim gehalten, lag es innerhalb eines Sperrgebiets auf dem Gelände der UHA Hohenschönhausen und war für die Insassen der 17 UHA des MfS sowie für die Häftlinge des Lagers X zuständig (ebd.: 77). Die Gründe für die Einlieferung der nach gegenwärtigem Stand zwischen 1959 und 1989 stationär aufgenommenen 3,173 Häftlinge waren formal vor allem die Gewährleistung beziehungsweise Herstellung von deren Haft‑, Prozess- und Vernehmungsfähigkeit (Spohr 2015: 227). Nach dem Ausbau der Einrichtung im Jahr 1977 arbeiteten im Komplex des Haftkrankenhauses circa 50 hauptamtliche Mitarbeiterinnen und Mitarbeiter (26 davon gehörten dem Wachpersonal an). Mit der rasanten Expansion des MfS – von 4,500 hauptamtlichen Mitarbeitern 1950 auf über 90.000 im Jahr 1989 – wuchs auch der medizintechnische und psychiatrische Verantwortungsbereich innerhalb der Haftanstalten. Das HKH bildete eine interne medizinische Infrastruktur, die von der Röntgendiagnostik über Zahnheilkunde bis zur Leichenkammer reichte, aber zugleich strikt konspirativ operierte. Der ärztliche Dienst war – anders als in regulären Krankenhäusern der DDR – weitgehend in die operative Logik der Hauptabteilung IX eingebunden.

Unterhalb der Leitungsebene gab es zwei Fachärzte, einen Zahnarzt sowie einen Facharzt für Psychiatrie und Neurologie.^3^ Die ständige Beschäftigung eines Facharztes für Psychiatrie und Neurologie ab 1978 deutet bereits auf einen Schwerpunkt der medizinischen Arbeit im Haftkrankenhaus hin. Obwohl angeschossene „Grenzverletzer“ dort regelmäßig eingeliefert und konspirativ versorgt wurden, kam der Großteil der Häftlinge aufgrund von sogenannten Haftreaktionen wie „Panikattacken“ oder „Haftpsychosen“ in die Einrichtung. Sie wurden psychiatrisch beobachtet und in der Regel medikamentös behandelt (Erler 2015: 77). Dass solche Medikationen auch darauf abzielten konnten, Geständnisse zu erpressen und während der Vernehmungen Informationen zu gewinnen, war stets vermutet worden. Das ließ sich bislang jedoch weder juristisch noch wissenschaftlich erhärten. Erst jüngste Forschungen haben erstmals Hinweise dafür in den Krankenakten gefunden (Wickert et al. 2026). Auch wurden bisweilen im Haftkrankenhaus selbst Vernehmungen durchgeführt (Spohr 2015: 229). Zum Aufgabenbereich des Psychiaters gehörte die forensische Analyse von Beweismitteln, das Erstellen von Täterhypothesen sowie die forensisch-psychiatrische Begutachtungen von Gefangenen. Vor allem dieser Tätigkeitsbereich verlieh dem Psychiater eine herausragende Stellung im Gefüge von Haftkrankenhaus und Haftanstalt. Seine Arbeit gewährte zwangsläufig tiefe Einblicke in Einzelfälle, setzte eine enge Absprache mit der Untersuchungsabteilung (Linie IX) voraus und war in die konspirativ brisante „Außenkommunikation“ des MfS mit der Staatsanwaltschaft und anderen Gerichtsakteuren eingebunden. Neben diesen Erkenntnissen über die institutionellen Strukturen ist unklar, inwieweit das Personal des Haftkrankenhauses zur geheimpolizeilichen Praxis während der Untersuchungshaft beigetragen hat. Dies liegt nicht nur an fehlenden schriftlichen Überlieferungen, sondern auch an den Aussagen ehemaliger Mitarbeiterinnen und Mitarbeiter des Haftkrankenhauses. Diese haben die Vorwürfe ehemaliger Häftlinge über den Einsatz von Medikamenten zu Verhörzwecken stets zurückgewiesen. So versucht Horst Böttger, der bis 1988 als Psychiater im Dienstrang eines Offiziers im Haftkrankenhaus tätig war, ein Bild von einer rechtlich einwandfreien Gefängnismedizin zu vermitteln, die keinerlei Berührungspunkte mit geheimpolizeilichen Anforderungen und Praktiken aufgewiesen habe:

„Unsere Medikation war auf das wirklich Notwendige beschränkt. Wahrheitsdrogen standen uns nicht zur Verfügung. Die Verwendung von Medikamenten erfolgte in Absprache mit dem Patienten. Der Zweck der Behandlung wurde erläutert. Eine derart intensive Auskunftspflicht, wie sie heute oft in schriftlicher Form und mit Unterschrift der Beteiligten praktiziert wird, gab es damals noch nicht. Eine Aufklärung erfolgte im Sinne einer Erklärung der als notwendig angesehenen Medikation und eventuell auftretender Probleme. Diese medizinischen Zusammenhänge waren auch Teil der Information an den Staatsanwalt“ (Böttger 2022: 103).

**Abb. 1:**
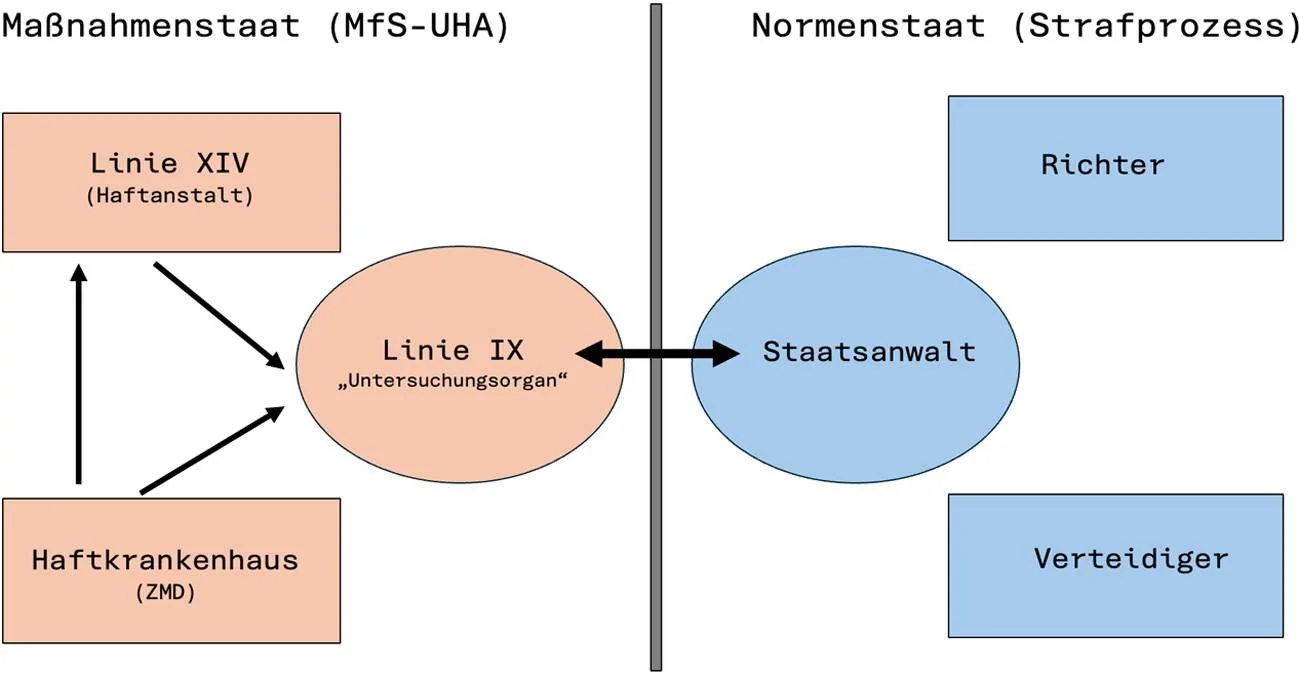
Zusammenwirken der für die Untersuchungshaft relevanten MfS-Linien und die Beziehung des MfS zu den Gerichtsakteuren im politischen Strafprozess der DDR, dargestellt am Beispiel der UHA Berlin-Hohenschönhausen (*Quelle*: Tilman Wickert)

Zutreffend an der Aussage ist sicherlich, dass die „medizinischen Zusammenhänge“ – beziehungsweise deren schriftliche Dokumentation – Teil der formalen Kommunikation des MfS mit Staatsanwaltschaft und Gerichten waren. Diese Überlieferungsebene besitzt einen hohen Quellenwert, da sie es erlaubt, die mögliche Doppelfunktion medizinischer Maßnahmen zu analysieren. So lässt sich prüfen, ob Medikationen ausschließlich der gefängnismedizinischen Versorgung dienten oder zugleich in strategische Überlegungen der Staatssicherheit eingebettet waren – etwa zur Beeinflussung von Häftlingen in der Untersuchungshaft. Damit rückt auch die organisatorische Logik des MfS in den Fokus, insbesondere das Zusammenspiel seiner einzelnen Diensteinheiten („Linien“) untereinander sowie mit den Gerichtsakteuren im Strafprozess. Für die Betroffenen verschwammen medizinische Behandlung, Überwachung und Vernehmungsdruck oftmals zu einer einzigen repressiven Erfahrungslandschaft. Tatsächlich jedoch unterstanden Haftunterbringung, Vernehmungen und medizinische Versorgung unterschiedlichen Dienstabteilungen der Stasi. Die UHA selbst, einschließlich der als Verwahrräume bezeichneten Zellen, gehörten zur Linie XIV. Die stationäre Versorgung von Gefangenen fand gegebenenfalls zentral im Berliner Haftkrankenhaus statt, das dem ZMD unterstand. Die Verhöre der Häftlinge erfolgten durch die Mitarbeiter der Linie IX. „Die Neun“ hatte in diesem Gefüge eine besondere Stellung. Als Untersuchungsorgan des MfS war sie bei den Untersuchungsvorgängen federführend. Ihren Untersuchungsführern (den Vernehmern) kam die Aufgabe zu, mit den Häftlingen Geständnisse „zu erarbeiten“ und nachrichtendienstlich relevante Informationen abzuschöpfen (Passens 2012: 27–35). Als „Hauptabteilung“ unterstand die Linie IX direkt dem Minister für Staatssicherheit Erich Mielke. Sie war zudem weisungsbefugt gegenüber den beigeordneten Abteilungen wie der XIV oder dem ZMD. Wie die Unterbringung der Gefangenen in den Zellen oder im Haftkrankenhaus im Einzelnen gestaltet wurde, bestimmten die Untersuchungsführer der Linie IX sowie ihre Referatsleiter, mit denen die Untersuchungsführer die Vernehmungsstrategien im Einzelfall abstimmten.^4^ Darüber hinaus war die Linie IX für die Außenkommunikation des MfS mit den Staatsanwaltschaften verantwortlich und somit für den Austausch mit den Strafprozessakteuren, die sich außerhalb der geheimpolizeilichen Sphäre befanden. Daraus ergab sich für die Linie IX die Aufgabe, Beweise für eine vermeintliche Straftat zu „offizialisieren“, um in einem formal rechtsstaatlichen Gerichtsverfahren bestehen zu können. Andererseits mussten sämtliche geheimpolizeilichen Instrumente – wie Druck in den Verhören, der Einsatz von Inoffiziellen Mitarbeitern (sogenannten IM), Zellenspitzeln sowie das Abhören von Telefonen oder Anwaltsgesprächen – vor der Gerichtsöffentlichkeit verborgen bleiben, sodass die Handschrift der Stasi bei der Manipulation der „Beweisführung“ im Vorfeld des Strafprozesses nicht erkennbar war (Booß & Richter 2024: 107). Somit agierte und bewegte sich die Linie IX als einzige Abteilung des MfS wechselseitig in und zwischen den zwei staatlichen Sphären einer Diktatur: zum einen in der maßnahmenstaatlichen Sphäre – dem natürlichen Wirkungsraum einer Geheimpolizei unter den Bedingungen einer Diktatur (Abb. 1). Die hier eingesetzten Maßnahmen dienten ausschließlich der Herrschaftssicherung, bewegten sich außerhalb des geschriebenen Rechts und waren daher für die Bürgerinnen und Bürger weder sichtbar noch genau kalkulierbar. Zum anderen fungierte sie als „Untersuchungsorgan“ im Strafprozess und trat somit auch in die normenstaatliche Sphäre ein, in der sich das SED-Regime der Öffentlichkeit als Rechtsstaat inszenierte und alles einer formal korrekten Strafprozessordnung zu folgen schien.^5^

Konkret bedeutete dies, dass die Linie IX in der schriftlichen Kommunikation mit der Staatsanwaltschaft eine Art Filterfunktion übernahm. Alle Informationen zu Maßnahmen, die das MfS selbst als geheimpolizeiliche Instrumente verstand, mussten ausgesondert und zurückgehalten werden. Dieses Vorgehen erwies sich bisweilen als fehleranfällig, sodass es immer wieder vorkam, dass der Staatsanwaltschaft unbeabsichtigt Einblicke in die geheimpolizeilichen Praktiken gewährt wurden (Booß 2017: 553). Beispielhaft lässt sich die Interaktion zwischen MfS und Gerichtsakteuren am Ablauf eines Gutachterverfahrens verdeutlichen: Beantragte ein Verteidiger eine medizinische Einschätzung oder ein forensisch-psychiatrisches Gutachten, richtete er dieses Gesuch an die Staatsanwaltschaft. Von dort wurde das Anliegen an die Linie IX weitergeleitet, die wiederum die Leitung des Haftkrankenhauses zur Erstellung eines entsprechenden Gutachtens anwies. Eine direkte Kommunikation zwischen Verteidigung, Staatsanwaltschaft und Haftkrankenhaus fand dabei nie statt. Die Linie IX fungierte stets als zwischengeschaltete Instanz, die den Kommunikationsfluss kontrollieren, filtern und im eigenen Sinne steuern konnte. Lag das Gutachten schließlich der Staatsanwaltschaft vor, wurde das Haftkrankenhaus in den Unterlagen bewusst verschleiert – etwa durch die Legende einer „Sektion Forensik der Humboldt-Universität“ oder eines „Krankenhauses des Ministerrates in Berlin-Buch“. Auf diese Weise wurde der tatsächliche Entstehungsort systematisch vor der Gerichtsöffentlichkeit verborgen.

## Erkenntnisse: Fahndung nach dem „Hobby-Anwalt“ „Clausmann“

Der Fall „Clausmann“ stellt einen jener Vorgänge dar, in denen das MfS mangels strafrechtlicher Substanz zunehmend auf Konstruktionen zurückgriff. Als die Staatssicherheit die Identität eines Mannes ermittelte, der mehrfach Ausreisewilligen Hinweise gab und rechtliche Schritte erläuterte, stand fest, dass „Clausmann“ keine illegalen Handlungen begangen hatte. Sein Verhalten widersprach jedoch der Logik des Systems. Er machte Bürgerinnen und Bürger mündig gegenüber einem Staat, der Passivität verlangte. Der aus diesem Grund eingeleitete operative Vorgang wurde mit äußerster Diskretion durchgeführt. Im Herbst 1985 erhielt die MfS-Kreisdienststelle in einer Kleinstadt im Bezirk Halle Informationen über folgenden Sachverhalt: Ein unbekannter Einwohner stand im Verdacht, als „Hobby-Anwalt“ seine Mitbürgerinnen und Mitbürger systematisch in Ausreiseangelegenheiten zu beraten und Hilfestellung bei der Formulierung von Ausreiseanträgen zu leisten.^6^ Auf den ersten Blick erschien dies als Routineangelegenheit, denn der Kampf gegen die Flucht- und Ausreisebewegung war in den 1980er Jahren eine der Hauptaufgaben des MfS. Allein 70 bis 80 Prozent der Ermittlungsverfahren, die die Linie IX in dieser Zeit „untersuchte“, betrafen solche Vorgänge (Passens 2012: 313–314). Anträge auf ständige Ausreise beziehungsweise auf Entlassung aus der DDR-Staatsbürgerschaft – nicht zu verwechseln mit kurzfristigen Reiseanträgen aus familiären Gründen – waren nach geltendem DDR-Recht nicht illegal. Doch nach der Unterzeichnung der KSZE-Akte von 1975, in der sich die DDR-Regierung international zur Einhaltung der Menschenrechte und zur Freizügigkeit ihrer Bürgerinnen und Bürger bekannt hatte, stieg die Zahl solcher Anträge sprunghaft an. Dieses wachsende gesellschaftliche und politische Problem wurde bald auch zu einem zentralen Handlungsfeld des MfS. Da die Antragstellung selbst nicht strafbar war, musste die Stasi bei der Bekämpfung „hartnäckiger Antragsteller“ auf indirekte Methoden zurückgreifen. Es war notwendig, die Betroffenen mithilfe geheimdienstlicher Verfahren in ein strafrechtlich verwertbares Licht zu rücken, um sie auf indirekte Weise zu kriminalisieren. Dieses Vorgehen wurde durch die Strafrechtsänderungsgesetze von 1977 und 1979 gestützt. Mit diesen Gesetzen verschärfte die SED gezielt einige der „Gummiparagrafen“, um die Ausreiseproblematik anzugehen (Raschka 2009: 69–71). Antragsstellerinnen und Antragsteller, die zum Beispiel im Vorfeld zu Informationszwecken die Ständige Vertretung der Bundesrepublik aufgesucht hatten oder sich mit ihrer Westverwandtschaft brieflich über das Vorgehen absprachen, konnten nun wegen „ungesetzlicher Verbindungsaufnahme“ oder wegen des „Sammelns oder Verbreitens von Nachrichten“, die geeignet waren „die Interessen der DDR zu schädigen“ (§ 219 StGB), angeklagt und inhaftiert werden. Wer wiederum Verzögerungen oder Ablehnungen der Anträge öffentlich als politisch motiviert oder durch das Wirken des MfS verursacht kritisierte, lief Gefahr, wegen „öffentlicher Herabwürdigung“ (§ 220 StGB) staatlicher Organe und Funktionsträger zu Haftstrafen von bis zu zwei Jahren verurteilt zu werden. Auf diese Weise erhielt das MfS ein Instrumentarium, mit dem es gezielt Ausreiseantragstellerinnen und Ausreiseantragsteller verfolgen konnte und das zugleich den Anschein eines rechtsstaatlich fundierten Vorgehens wahrte.

Nach einigen Monaten und weiteren Verhören von Gefangenen sowie dank der Informationen eines IM ermittelte das MfS schließlich die Identität des kleinstädtischen „Hobby-Juristen“. Es handelte sich um einen knapp 40 jährigen Ingenieur und verheirateten Familienvater. Umgehend schaltete sich der geheimpolizeiliche Apparat scharf. Das MfS leitete einen Operativen Vorgang mit dem Decknamen „Inspirator“ ein und überwachte „Clausmann“ permanent. Rasch stellte das MfS fest, dass man es hier nicht mit einem üblichen Ausreisedelikt zu tun, sondern eine veritable Ein-Mann-NGO ins Visier genommen hatte. „Clausmann“ betrieb nicht etwa Freundschaftsdienste oder Nachbarschaftshilfe, sondern ging seine Anliegen systematisch an. Er trug Verordnungen und einschlägige Presseberichte zur Ausreiseantragstellung zusammen und verwies Ausreisewillige an einen ihm bekannten Rechtsanwalt. Zudem nahm er an Beratungsgesprächen teil und fertigte Notizen an, die er für eigene Konsultationen nutzte.^7^ Bis zum Frühjahr 1986 hatte er fünf Personen in Ausreiseangelegenheiten unterstützt – teilweise mit Erfolg, wie die Überwachung durch das MfS belegt. Mit zwei seiner „Mandanten“ unterhielt er Briefkontakt in die Bundesrepublik. Der Aufbau einer derartigen Bürgerrechtsberatungsstelle, noch dazu in Verbindung mit einem Anwalt, der als Bevollmächtigter der Kanzlei von Wolfgang Vogel^8^ in Berlin arbeitete, war dem MfS ein Dorn im Auge. Allerdings waren diese Aktivitäten juristisch nicht strafbar. Nach Gesetzgebung der DDR fehlte es an einer relevanten Grundlage für eine Anklage.

Um „Clausmann“ dennoch kriminalisieren zu können, griff das MfS zu einer bekannten Strategie: der Einbindung bereits verurteilter Personen, die operativ zugänglich waren. Im konkreten Fall waren dies die Eheleute „Müller“, die 1985 wegen eines Briefs an den bayerischen Ministerpräsidenten Franz Josef Strauß im Rahmen ihres „Übersiedlungsgesuches“ zu Haftstrafen verurteilt worden waren. Noch während ihrer Haft wurden sie 1986 erneut vom MfS befragt und gaben an, „Clausmann“ habe ihnen bei der Abfassung eines Briefs geholfen, um ihre Ausreise zu beschleunigen.^9^ Der Fall der Familie „Müller“ verdeutlicht die Vorgehensweise des MfS im Umgang mit „hartnäckigen Antragstellern“ und veranschaulicht die Herrschaftspraxis der Staatssicherheit. Aus diesem Grund wird er hier näher skizziert. Die Eheleute „Müller“ verkörpern jenes typische Profil von Bürgerinnen und Bürgern der DDR, die Mitte der 1980er Jahre ein Übersiedlungsgesuch stellten. Beide waren Industriearbeiter mittleren Alters, verheiratet und in einer Kleinstadt wohnhaft. Politisch waren sie unauffällig, gehörten weder der SED noch kirchlich-oppositionellen Kreisen an.^10^ Ihr 1984 gestellter Antrag auf dauerhafte Ausreise war formal zulässig, politisch jedoch unerwünscht. Ein Brief an ihre Tante in West-Berlin, verbunden mit der Bitte um Unterstützung durch Franz Josef Strauß, wurde durch die Postkontrolle der Staatssicherheit abgefangen und löste eine mehrstufige Bearbeitung aus.^11^ Die operativen Maßnahmen, zu deren Durchführung drei IM im Umfeld der Eheleute „Müller“ mobilisiert wurden, folgten dem bekannten dreistufigen Schema des MfS: Eindämmung, Zurückdrängung, Kriminalisierung.^12^ Im Rahmen der Eindämmung sollten die beiden davon abgehalten werden, andere Mitbürger zu Ausreiseanträgen zu inspirieren. Die Zurückdrängung sah vor, die Eheleute „Müller“ zur Rücknahme des Übersiedlungsgesuch zu bewegen. Letzte Maßnahme war ihre offene Kriminalisierung. Hier sah der Plan vor, dem Ehepaar „Müller“ eine Straftat nach § 219 StGB (Verbreiten von Nachrichten, die geeignet sind, die Interessen der DDR im Ausland zu schädigen) nachzuweisen.^13^ Zur Eindämmung wurden jedoch keine signifikanten Maßnahmen eingeleitet, da die Eheleute weder andere zur Antragstellung inspiriert noch zur Solidarisierung anregt hatten. Im Gegenteil: Wie viele Antragsteller betrachteten sie ihre Ausreisepläne als Privatangelegenheit, wichen Gesprächen darüber aus und hielten diese auch ihrem 8‑jährigen Sohn gegenüber geheim.^14^ Im Rahmen der Zurückdrängung wies das MfS die Volkspolizeidienststelle an, Herrn „Müller“ den Personalausweis zu entziehen und ihm stattdessen einen vorläufigen PM12-Ausweis auszuhändigen, den verurteilte Straftäter mit sich zu führen hatten^15^. Diese Maßnahme wurde von ehemaligen Inhaftierten in Oral-History-Interviews als stark stigmatisierend beschrieben (Söhner et al. 2025, 2026).

Die Vorgesetzten und Kollegen im Betrieb der Eheleute „Müller“ fügten sich hingegen nicht umstandslos in die schikanösen Zurückdrängungsmaßnahmen der Stasi ein. Die Funktionäre der Betriebsleitung luden die Eheleute zwar getrennt zu Aussprachen vor, jedoch nicht in der vom MfS gewünschten Häufigkeit. Überdies hielt die Leitung es für „nicht angemessen“, das Arbeitskollektiv gegen diese in Stellung zu bringen und sprach dem Kollektiv rundweg die Eignung für derlei politische Einflussnahme ab.^16^ Nur auf Insistieren des MfS wurde Herr „Müller“ schließlich doch von seinem Arbeitsplatz an einer Werkzeugmaschine abgezogen, obwohl die Betriebsleitung dies mit Verweis auf seine Kompetenzen als Maschinenführer anfänglich abgelehnt hatte.^17^ Das MfS zeigte sich sichtlich verärgert. Die Genossen der Betriebsleitung wären „sich ihrer Verantwortung in der Arbeit mit Übersiedlungsersuchenden nicht bewusst“ und nicht bereit, „sich mit diesen […] mehr als nötig auseinanderzusetzen“.^18^ Die Zurückdrängungsmaßnahmen liefen ins Leere. Die Eheleute „Müller“ beharrten auf ihrem Übersiedlungsersuchen mit Verweis auf die Zugeständnisse der DDR-Regierung bei Familienzusammenführungen im zuvor unterzeichneten Madrider Abkommen.^19^ Überdies beschwerten sie sich schriftlich beim Ministerium des Innern und beim Staatsrat der DDR in Ost-Berlin über die schikanöse Einziehung von Herrn „Müllers“ Personalausweis und die Einmischung der Parteien in ihr Privatleben durch die aufgenötigten Gespräche mit der Betriebsleitung.^20^

Auch die umfangreichen technischen Überwachungsmaßnahmen des MfS in der Familienwohnung blieben unergiebig. Bei zwei konspirativen Durchsuchungen dokumentierte das MfS zwar die Korrespondenz von Frau „Müller“ mit ihrer Tante in West-Berlin fotografisch, darunter auch den Brief an Strauß. Doch diese waren – im Gegensatz zu den Durchsuchungen des MfS – nicht gesetzeswidrig und stellten keinen Straftatbestand dar.^21^ Folglich konnten sie nicht „offizialisiert“ und vor Gericht verwendet werden. Auch die Abhörung der Wohnung brachte keine strafrechtlich verwertbaren Ergebnisse, lieferte jedoch der Stasi erstmalig Informationen über die juristische Beratung der „Müllers“ durch eine unbekannte Person.^22^

Vor dem Hintergrund der erfolglosen Maßnahmen modifizierte das MfS seine Strategie und setzte einen „Offizier im besonderen Einsatz“ (OibE; vgl. Flach & Dreke 2013) im Rat der Stadt gegen die Eheleute „Müller“ ein, der als Verwaltungsmitarbeiter der Abteilung Inneres für solche Fälle zuständig war. Im Juni 1985 informierte dieser Herr „Müller“ erneut über die Ablehnung seines Übersiedlungsgesuchs. Laut dem MfS prophezeite „Müller“ daraufhin, dass die Verwandte in West-Berlin nun „Aktivitäten entwickeln werde, die für die DDR mit außenpolitischen Problemen verbunden wären“.^23^ Aufgrund dieser vermeintlich getätigten, von Herrn „Müller“ aber später bestrittenen Aussagen leitete das MfS im Juni 1985 ein Ermittlungsverfahren nach § 214 StGB („Beeinträchtigung staatlicher oder gesellschaftlicher Tätigkeit“) ein und inhaftierte ihn in der UHA Potsdam.^24^

In den folgenden Wochen verhörte die Abteilung IX, das Untersuchungsorgan der Stasi, ihn mehrfach. Ziel war es, ein Geständnis nach § 214 StGB zu erlangen und Informationen darüber zu gewinnen, welche bundesdeutschen Stellen die Tante in West-Berlin zur Unterstützung des Ausreiseantrags kontaktiert hatte.^25^ Die genauen Methoden, mit denen die Vernehmungsoffiziere der Linie IX Herrn „Müller“ unter Druck setzten, sind nicht mehr rekonstruierbar, da die Protokolle vernichtet wurden.^26^ Schließlich gelang es ihnen, einen Straftatbestand nach § 219 zu konstruieren und die Ermittlungen auf Frau „Müller“ auszudehnen.^27^ Der Rat der Stadt verfügte die Einweisung ihres 8‑jährigen Sohnes in ein Heim.^28^ Um dies zu verhindern, erteilte Frau „Müller“ am Tag ihrer Verhaftung einer befreundeten Familie schriftlich eine Fürsorgevollmacht für ihren Sohn.^29^ Die Familie lehnte jedoch eine vorübergehende Inobhutnahme mit dem Verweis auf beengte Wohnverhältnisse ab, nachdem ein stellvertretender Leiter der MfS-Kreisdienststelle persönlich auf sie zugekommen war, um das „Problem bezüglich der Übernahme des Sohnes“ zu „erläutern“.^30^ Der Sohn wurde daraufhin in ein Kinderheim verbracht. Seine Eltern wurden im September 1985 wegen § 219 StGB zu einem Jahr und vier Monaten (Frau „Müller“) und wegen § 214 StGB in Tateinheit mit § 219 StGB zu einem Jahr und acht Monaten (Herr „Müller“) Haft verurteilt. Ihr Fall lieferte das operative Fundament, auf dem die Kriminalisierung „Clausmanns“ aufgebaut wurde. Da die Eheleute „Müller“ gegenüber dem MfS die Unterstützung „Clausmanns“ bei der Abfassung des Briefes an F. J. Strauß bekannt hatten, erhob das MfS den Vorwurf der „ungesetzlichen Verbindungsaufnahme“ (§ 219 StGB) auch gegenüber „Clausmann“ und inhaftierte diesen Anfang April 1986 in der UHA des MfS in Halle.^31^

## Der Versuch der Kriminalisierung „Clausmanns“

In den Wochen nach seiner Verhaftung ahnte die Stasi, dass ihre Kriminalisierungsstrategie trotz operativer Einbindung der Eheleute „Müller“ zu scheitern drohte. „Clausmann“ widersprach beharrlich dem Vorwurf, bei der Formulierung des Briefs an Strauß mitgewirkt zu haben und verweigerte ein Geständnis.^32^ Stattdessen verwies er wiederholt auf die Unrechtmäßigkeit seiner Inhaftierung und betonte, er habe mit seinen Beratungstätigkeiten ausschließlich darauf hingewirkt, dass die Ausreiseanträge im Einklang mit den geltenden Gesetzen der DDR formuliert wurden. Die Aussagen der Ehepartner „Müller“ waren bezüglich seiner Rolle zudem widersprüchlich. Dienstpläne der „Müllers“ widerlegten, dass er am besagten Tag den Brief diktiert haben konnte.^33^ Auch die Laboranalyse der bei der Hausdurchsuchung sichergestellten Kaminasche zeigte keine Rückstände verbrannter Briefe.^34^ Darüber hinaus verliefen die Abhörmaßnahmen in „Clausmanns“ Zelle ohne verwertbare Ergebnisse.^35^ Der Ermittlungsdruck blieb für die Stasi hoch. Zugleich zeigen die Überlieferungen, dass sich das MfS nicht nur bei den Ermittlungen schwertat, sondern sich auffällig zurückhielt – offenbar um Aufmerksamkeit zu vermeiden. So unterließ die Stasi die Durchsuchung der Büroräume an „Clausmanns“ Arbeitsplatz in einem großen Volkseigenen Betrieb, während seine Privatwohnung mit äußerster Akribie durchsucht wurde. Auch der ursprüngliche Plan, in seinem Wohn- und Arbeitsumfeld IM zu installieren, wurde nicht weiterverfolgt. Dies weist auf die bekannten Schwierigkeiten der Stasi bei der Führung von IM in Kleinstädten unterhalb der Leitungsebenen hin (Hürtgen 2014: 23–24). Vor diesem Hintergrund drohte im Mai 1986 das Vorhaben zu scheitern, „Clausmann“ wegen des Verstoßes gegen § 219 StGB strafrechtlich zu belangen. Darauf reagierte das MfS mit einem Strategiewechsel und erweiterte die Anklage um § 220 StGB, der die „öffentliche Verleumdung“ und „Herabwürdigung“ Staatlicher Organe unter Strafe stellte.^36^ Grundlage hierfür bildeten „Clausmanns“ Aussagen bei einer Gegenüberstellung mit dem Ehepaar „Müller“. Er hatte sachlich korrekt die Unrechtmäßigkeit seiner Inhaftierung durch das MfS kritisiert. In Reaktion auf die Äußerung der Staatsanwältin, er sei an seiner Situation „selbst schuld“, wies er auf ihre Befangenheit hin, da die Schuldfrage noch nicht geklärt sei.^37^ Ferner unterstellte er dem MfS Foltermethoden aufgrund der fehlenden orthopädischen Behandlung in der UHA Halle.^38^ Das MfS wertete diese Aussagen als „öffentliche Herabwürdigung“ des Untersuchungsorgans und der Staatsanwaltschaft und begründete damit die Erweiterung der Anklage um § 220 StGB. Die nach dem Gesetz notwendige „Öffentlichkeit“ war nach Ansicht der Stasi durch die Anwesenheit von Frau „Müller“ hergestellt. Dass die Eheleute „Müller“ sich in diese Strategie einfügten und damit ihrem Ratgeber schadeten, ist vor dem Hintergrund ihrer persönlichen Situation nachvollziehbar. Beide waren wegen ihrer hartnäckigen Ausreisebemühungen inhaftiert und ihr gemeinsamer Sohn in einem Kinderheim untergebracht worden. Schließlich erhielt die Familie die Zusage, über das Stasi-Gefängnis Kasberg in die Bundesrepublik ausreisen zu dürfen.^39^ Zuvor mussten sie jedoch in der UHA Halle gegen „Clausmann“ aussagen, wie Herr „Müller“ einem Freund in einem offenbar nicht weitergeleiteten Brief berichtete.^40^ Dennoch benötigte das MfS für den Vorwurf gemäß § 220 StGB ein Geständnis oder zumindest ein Teilgeständnis von „Clausmann“. Die hierfür angesetzte Erweiterungsvernehmung fand nicht mehr in der UHA Halle, sondern im zentralen Haftkrankenhaus des MfS in Berlin-Hohenschönhausen statt. Die Überführung erfolgte am 4. Juni offiziell mit der Begründung eines Schulter-Arm-Syndroms und angeblich mit Einverständnis „Clausmanns“, der während seiner Haft in Halle über heftige Rückenschmerzen geklagt hatte, die sich auf eine Bandscheibenoperation wenige Monate zuvor zurückführen ließen.

## Medizinische Behandlung „Clausmanns“ im Haftkrankenhaus Berlin-Hohenschönhausen

Das Vorhaben, „Clausmann“ aufgrund seiner angeblichen Unterstützung bei dem Brief an den westdeutschen Politiker Strauß nach § 219 StGB strafrechtlich zu belangen, gestaltete sich zunehmend problematisch. Vor diesem Hintergrund gewann die geplante Erweiterungsvernehmung zum § 220 StGB am bevorstehenden 12. Juni besondere Bedeutung. Bereits die strategischen Vorbereitungen weisen darauf hin, dass die Vernehmer beabsichtigten, „Clausmann“ psychischem Druck auszusetzen. Der leitende Major veranlasste den zuständigen Untersuchungsführer sicherzustellen, dass der Besuch von „Clausmanns“ Ehefrau am 9. Juni unbedingt stattfinden sollte, obwohl der Häftling diesen aus Sorge um die seelische Belastung sowohl für sich als auch für seine Frau ablehnte.^41^ Es gelang aber, ihn zu überreden, und der Besuch fand statt. Der intendierte Effekt blieb jedoch aus. Statt einer Schwächung der Resilienz wurde „Clausmanns“ Kampfgeist mobilisiert. „Keine Spur mehr von der Depressivität der vergangenen Tage“ zeigte er nach dem Besuch seiner Frau, wie der Psychiater in der anschließenden Exploration feststellte.^42^ „Clausmann“ bestand auf seiner Unschuld, verwies auf die Rechtmäßigkeit seiner Beratungen, kritisierte die Überwachungsmaßnahmen der Stasi vor seiner Inhaftierung und bezichtigte führende Funktionäre seines Kreises der Korruption.^43^ Die Vernehmung seiner Frau im Anschluss an den Besuch blieb ebenfalls ergebnislos. Sie gab zu Protokoll, keine Kenntnisse über die Aktivitäten ihres Mannes zu haben, was sowohl den Aussagen der „Müllers“ als auch denen eines IM entsprach. Mit Verweis auf das geltende DDR-Recht wollte sie sich als Familienmitglied nicht weiter zu dem Fall äußern.^44^

**Abb. 2:**
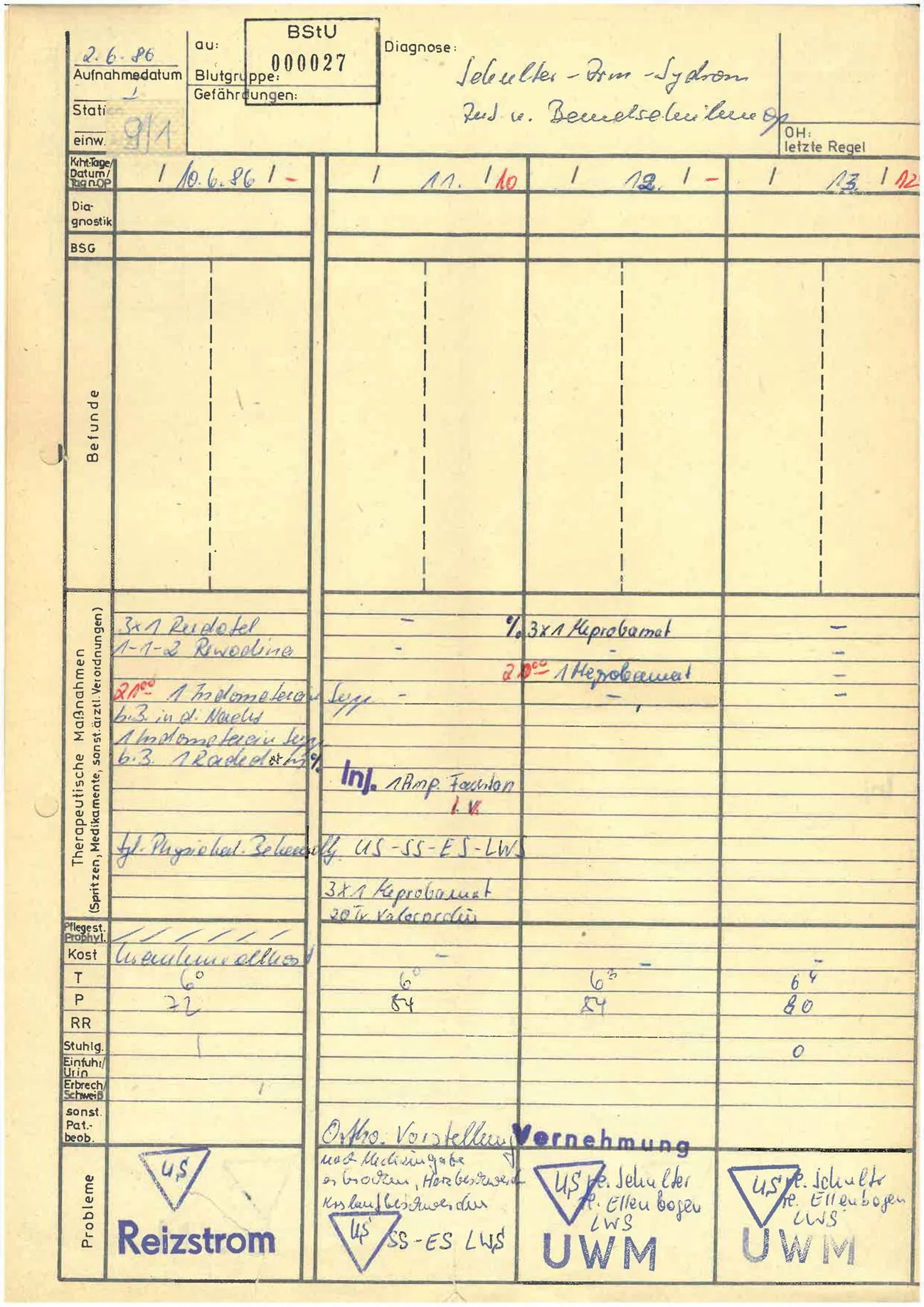
Auszug aus der Verlaufskurve aus „Clausmanns“ Krankenakte. (*Quelle*: Verlaufskurve in der Krankenakte von „Clausmann“, BArch MfS AS, Nr. 11249/89, Bl. 27)

Zu diesem Zeitpunkt zeichnete sich ab, dass die Kriminalisierungsstrategie des MfS zu scheitern drohte. Ein Zurückweichen – also eine Entlassung „Clausmanns“ – war jedoch nicht vorgesehen, sobald das MfS ein Ermittlungsverfahren eingeleitet hatte. Dieses fallspezifische Spannungsverhältnis zwischen dem hohem Kriminalisierungsdruck, dem Mangel an belastendem Beweismaterial und der Absicht, „Clausmann“ psychisch zu destabilisieren, bildet die Grundlage für das Verständnis der nachfolgenden Ereignisse. Für den 12. Juni um 8.15 Uhr setzte das MfS die Erweiterungsvernehmung an. Am selben Tag erhielt „Clausmann“ – vermutlich im Anschluss an die Vernehmung – sein orthopädisches Korsett zurück, das ihm in der UHA Halle noch vorenthalten worden war.^45^ In den 48 Stunden vor der Vernehmung wurden ihm sedierende Medikamente verabreicht, darunter verschiedene Tranquilizer wie Benzodiazepine und Histamine (Abb. 2). Die Kombination dieser Substanzen, zumal aufgrund ihrer unterschiedlichen Halbwertszeiten, konnte zu starker Müdigkeit, eingeschränkter Konzentrationsfähigkeit und Gedächtnisstörungen führen.^46^

Zur konkreten Dosierung enthalten die Unterlagen keine Angaben. In welchem Maße „Clausmann“ nach dieser Medikation am 12. Juni tatsächlich vernehmungsfähig war, kann retrospektiv daher nicht verlässlich rekonstruiert werden. Auffällig ist jedoch, dass er selbst im Rahmen eines zweiten Verhörs den Verdacht äußerte, seine Vernehmungs- und Aussagefähigkeit sei während der Erweiterungsvernehmung durch die verabreichten, ihm nicht bekannten Medikamente beeinträchtigt gewesen; er habe die Kontrolle über sein Aussageverhalten verloren.^47^ In der Folge widerrief „Clausmann“ seine Aussagen, verlangte ein Gespräch mit dem behandelnden Psychiater und forderte, die Tonbandaufnahmen der Erweiterungsvernehmung vorgespielt zu bekommen, um sich mit seinem Rechtsanwalt beraten zu können.^48^

Die Reaktionen der Stasi auf diese Vorwürfe sind besonders aufschlussreich im Hinblick auf die Frage, ob und in welcher Weise das MfS psychotrope Substanzen im Rahmen von Vernehmungen einsetzte. Umgehend wurde der Psychiater des Haftkrankenhauses hinzugezogen. Er beteuerte, es habe sich lediglich um Schmerzmittel gehandelt, die weder die geistigen Fähigkeiten beeinträchtigen noch am Tag der Erstvernehmung nachwirken würden.^49^ Beide Aussagen waren jedoch nachweislich falsch. Angesichts der bekannten Halbwertszeiten war eine kumulative Wirkung der verabreichten Substanzen höchst wahrscheinlich. Die in Abb. 2 dokumentierten Medikamente mussten vor allem in ihrer Wechselwirkung zu deutlichen kognitiven Einschränkungen führen, darunter Konzentrations‑, Aufmerksamkeits- und Gedächtnisstörungen. Die Vernehmungsprotokolle selbst legen nahe, dass „Clausmann“ während der Befragung unter Konzentrationsschwierigkeiten und Erinnerungslücken litt.^50^ Seine Forderung, ihm die Aufzeichnung der Erweiterungsvernehmung vorzuspielen und dem Gericht zur Verfügung zu stellen, wurde mit der Begründung zurückgewiesen, die Aufzeichnung hätte aus „technischen Gründen“ nicht erfolgen können –51 eine Erklärung, die die Ermittler wenig später auch gegenüber dem Staatsanwalt wiederholten, der sich das Tonband explizit „der Passage zum § 220“ wegen anhören wollte.^52^ Dass hierfür tatsächlich technische Ursachen vorlagen, erscheint äußerst unwahrscheinlich, da die Vernehmungsstrategie des MfS explizit vorsah, sämtliche Vernehmungen ohne Ausnahme auf Tonband zu dokumentieren.^53^ Noch aufschlussreicher ist ein weiteres Vorgehen: Die zuständige Untersuchungsabteilung des MfS, die Linie IX, wies die Leitung des Haftkrankenhauses schriftlich an, die Wirkungsweise der verabreichten Medikamente in den Gesundheitsunterlagen nicht zu vermerken. Diese Unterlagen sollten auf Antrag von „Clausmanns“ Rechtsanwalt der Staatsanwaltschaft vorgelegt werden. Wörtlich hieß es:

„Am 2.7. erfolgte die als Anlage beigefügte Anfertigung einer Einschätzung der Vernehmungsfähigkeit des obengenannten Beschuldigten. Nach Absprache mit dem Staatsanwalt wird gebeten, in Ergänzung des genannten Schreibens eine Aufstellung über die dem Beschuldigten C. verordneten Medikamente anzufertigen. Entsprechend der Forderung des Staatsanwaltes soll in dieser Aufstellung keine Angabe der Wirkungsweise der entsprechenden Medikamente enthalten sein.“^54^

**Abb. 3:**
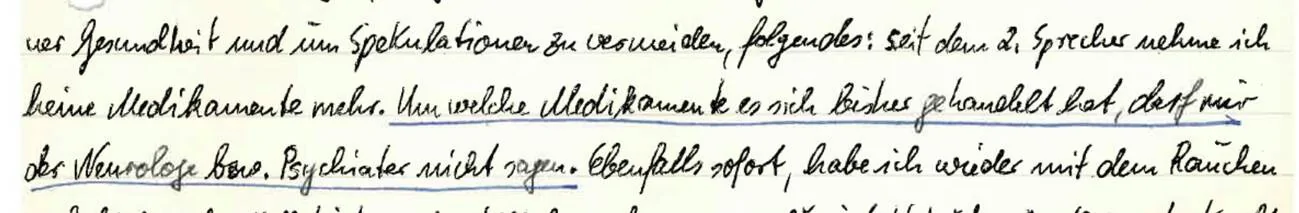
Auszug aus dem Brief „Clausmanns“ vom 11. Juli 1986 an seine Frau mit der Mitteilung: „Um welche Medikamente es sich bisher gehandelt hat, darf mir der Neurologe bzw. Psychiater nicht sagen“. Offenbar wegen dieser Passage, die das MfS mit Kugelschreiber unterstrichen hat, wurde der Brief einbehalten. (*Quelle*: Brief „Clausmanns“ an Frau vom 11. Juli 1986, BArch MfS BV Halle AU 840/87, Bd. 6 Bl. 100.)

Die Anweisung, die Wirkungsweise der Medikamente zu verschleiern, entsprach vorrangig dem Konspirationsbedürfnis der Linie IX, denn die Staatsanwaltschaft hatte dies in der genannten Absprache gar nicht gefordert.^55^ Allzu deutlich wird, dass das MfS die Auswirkungen der Medikationen auf die Erstvernehmung gezielt zu verbergen suchte. In dieses Muster fügt sich auch der Umgang mit einem Brief „Clausmanns“ an seine Frau ein. Darin beklagt er, der behandelnde Psychiater verweigere ihm jede Auskunft über die verabreichten Medikamente (Abb. 3). Das Schreiben wurde von der Linie IX abgefangen und nicht weitergeleitet.

Die Haftkrankenhausleitung richtete ihre Gesundheitsdokumentation also offenkundig an den strategischen Vorgaben der Linie IX aus. Die Wirkungsweise der Medikamente wurde in der Aufstellung bewusst nicht erwähnt. Auch der Haftfähigkeitsbericht bescheinigte „Clausmann“ zwar die volle Haftfähigkeit, ließ jedoch sowohl die relevanten Tage vom 10. bis 12. Juni als auch die Wirkung der eingesetzten Substanzen unerwähnt. Der Staatsanwaltschaft wurde lediglich mitgeteilt, die Behandlung sei „sowohl pharmakologisch als auch physiotherapeutisch kompliziert“ gewesen. Diese Schwierigkeiten erklärten die Ärzte jedoch nicht durch die Medikation, sondern schrieben sie „der psychischen Struktur“ des Häftlings zu. Entsprechend empfahlen sie nach der Hauptverhandlung eine „orthopädisch-psychiatrische Weiterbehandlung […] um eine anhaltende psychische Fehlverarbeitung des Beschwerdebildes zu verhindern“.^56^

Auch das Gerichtsurteil folgte den Vorgaben und strategischen Erwartungen der Stasi. Trotz der offenkundigen Widersprüche in den Zeugenaussagen und Ermittlungen des MfS, die der Verteidiger in einem für die DDR seltenen Freispruchplädoyer hervorgehoben hatte, wurde „Clausmann“ auf der Grundlage von § 219 und § 220 StGB zu einer Freiheitsstrafe von einem Jahr und neun Monaten verurteilt. Nach Verbüßung von sechs Monaten Haft wurde er in die Bundesrepublik entlassen.^57^

## Diskussion

Die Untersuchung des Falls „Clausmann“ erlaubt einen seltenen Einblick auf das Zusammenspiel von medizinischen Maßnahmen und operativer Ermittlungsstrategie im Haftkrankenhaus der Staatssicherheit. Die zentrale Frage, ob sedierende beziehungsweise bewusstseinsverändernde Medikamente gezielt als Bestandteil von Vernehmungstaktiken eingesetzt wurden, lässt sich nicht abschließend beantworten. Jedoch entsteht aus den archivalischen Befunden ein konsistentes Muster, das auf eine funktionale Komplizenschaft zwischen Medizinern und Vernehmern schließen lässt:

- Auffällig ist erstens die **zeitliche Koinzidenz**: Die Medikamentengabe erfolgte unmittelbar vor einer entscheidenden Erweiterungsvernehmung, in der ein belastbares Teilgeständnis für einen konstruierten § 220 StGB-Vorwurf erlangt werden sollte. Die gleichzeitig eingesetzten Substanzen – darunter Benzodiazepine und andere sedierende Wirkstoffe – besitzen Halbwertszeiten, die eine Beeinträchtigung der Konzentrations- und Merkfähigkeit sehr wahrscheinlich machen. „Clausmanns“ eigener Eindruck eines veränderten Bewusstseinszustands deckt sich mit dem pharmakologischen Profil. Die verwendeten Substanzen weisen pharmakologisch die Wirkprofile auf, die Inhaftierte in Interviews beschreiben – ein Muster, das als „institutionelle Koinzidenz“ bezeichnet werden kann, da es eine Übereinstimmung zwischen operativen Bedürfnissen und medizinisch-technischen Möglichkeiten darstellt.
  Die durch Interviews gewonnenen Hinweise, wie das „Wegtreten“ nach der Gabe von Faustan® (Diazepam) oder das auffällige Reden, deuten darauf hin, dass zumindest einige Inhaftierte pharmakologische Einflüsse als direkt mit den Verhören verbunden erlebten (Söhner et al. 2025). Die kategorischen Zurückweisungen Horst Böttgers, dem langjährigen Psychiater des Haftkrankenhauses, befinden sich in einem deutlichen Spannungsfeld divergierender Selbst- und Fremdwahrnehmungen.
- Zweitens weist die **selektive Dokumentation** auf eine operative Steuerung hin. In den für die Staatsanwaltschaft bestimmten Unterlagen fehlen Informationen zu den Wirkungen der eingesetzten Substanzen, und die Tonbandaufnahmen der entscheidenden Erweiterungsvernehmung vom 12. Juni wurden als „technisch nicht möglich“ deklariert und daher der Staatsanwaltschaft nicht übergeben. Auch die Weigerung, einen Brief weiterzuleiten, in dem „Clausmann“ seiner Ehefrau Details zur Medikation mitteilte, fügt sich in dieses Muster.
- Drittens zeigt sich ein **strategisch motiviertes Verschweigen** medizinischer Details durch den behandelnden Arzt. Die Anweisung der Linie IX an das HKH, die Wirkungsweise der Medikamente gegenüber der Staatsanwaltschaft zu verschweigen, stellt ein besonders klares Indiz dar. Sie deutet darauf hin, dass man sich der ermittlungstaktischen Relevanz der Medikation bewusst war und dass dieses Vorgehen in einem rechtsstaatlichen Rahmen nicht haltbar gewesen wäre. Es ist weniger der pharmakologische Eingriff selbst, der die Struktur offenbart, sondern vielmehr das kommunikative Management von Wissen, das auf operative Konspiration hinweist.

Ob die Medikation gezielt zu vernehmungstaktischen Zwecken verabreicht wurde oder das MfS ihre Wirkung nur billigend in Kauf nahm, geht aus den Akten nicht hervor. Ein gezielter Einsatz hätte jedoch in die ermittlungstaktischen Ziele der Stasi gepasst: Die Vernehmung vom 12. Juni sollte ein Geständnis oder zumindest ein verwertbares Teilgeständnis für den Vorwurf der „Öffentlichen Herabwürdigung“ hervorbringen – ein Vorwurf, den die Stasi als Ersatzkonstruktion eingeführt hatte, nachdem sich die Anklage nach § 219 StGB als unsicher erwiesen hatte. Da sich der inkriminierte Vorfall in Anwesenheit von Staatsanwaltschaft und MfS ereignet hatte, ging es nicht darum, neue Sachverhalte aufzudecken, sondern darum, „Clausmann“ zu Aussagen zu bewegen, die seine Kritik als strafwürdige Herabwürdigung erscheinen ließen. Gerade weil er sich zuvor als argumentationsstark und rechtlich trittsicher erwiesen hatte, konnte der Einsatz mehrerer sedierender Substanzen ermittlungstaktisch als Mittel gesehen werden, seine kognitive Leistungsfähigkeit zu schwächen und ihn in eine argumentative Defensive zu drängen. Damit erhoffte sich das MfS offensichtlich einen entscheidenden ermittlungstaktischen Vorteil für die „Erweiterungsvernehmung“, um vor Gericht eine belastbare Grundlage für den § 220 StGB zu schaffen.

Der Fall „Clausmann“ belegt kein flächendeckendes Medikamentenprogramm, sondern einen situativen, opportunistischen Einsatz medizinischer Ressourcen, wenn operative Ziele anders nicht zu erreichen waren. Die Einbindung medizinischer Ressourcen in die Ermittlungslogiken des MfS schuf die Voraussetzungen, unter denen dies möglich wurde. Die Befunde deuten darauf hin, dass das HKH – zumindest im Fall „Clausmann“ – nicht als unabhängige medizinische Instanz agierte, sondern als operativer Resonanzraum der Stasi, der medizinische Maßnahmen gezielt einsetzte und in eine konspirativ geführte Ermittlungsstrategie integrierte. Hierbei steht weniger der Nachweis eines konsistenten „Medikamentenprogramms“ im Vordergrund, sondern vielmehr die strukturelle Möglichkeit und punktuelle Nutzung medizinischer Handlungen zur Vorbereitung und Stabilisierung politisch motivierter Strafverfahren. Vor dem Hintergrund der eingangs skizzierten Strukturen und Arbeitsweisen der MfS-Linien sowie der Außenkommunikation der Stasi mit der Staatsanwaltschaft liegt es daher nahe, die Medikation in den Tagen vor der Vernehmung als geheimpolizeiliches Instrument zur Geständnisgewinnung zu interpretieren. Der konspirative Umgang mit der Medikation seitens des MfS gegenüber der Staatsanwaltschaft entspricht dessen Vorgehen bei Maßnahmen, die als geheimpolizeiliche Instrumente galten und aufgrund ihrer Rechtswidrigkeit strengstens abgeschirmt werden mussten. Folgt man dieser Logik, lässt sich der Einsatz von psychotrop wirkenden Medikamenten als integraler Bestandteil der Vernehmungsstrategie der Linie IX im Fall „Clausmann“ und möglicherweise darüber hinaus verstehen.

Die Interviews mit ehemaligen Häftlingen unterstreichen dieses Bild (Söhner et al. 2025, 2026). Die wahrgenommenen Bewusstseinseinschränkungen, die fehlende Aufklärung über Medikamente, deren ritualisierte Übergabe durch das Wachpersonal – all dies verweist auf ein System, in dem medizinische Praktiken nicht primär therapeutischen Zwecken dienten, sondern einem Dispositiv der Kontrolle, Einschätzung und Steuerung. Gleichzeitig verdeutlichen die Aussagen ehemaliger Mitarbeiter, die einen Missbrauch vehement bestreiten, die typischen Spannungen zwischen ärztlicher Selbstwahrnehmung und Betroffenenberichten in autoritären Systemen. So präsentiert die Autobiografie des Psychiaters Böttger ein klassisches Verteidigungsnarrativ: professionelle Integrität, politisches Missverständnis, keine voluntative Überschreitung (Böttger 2022). Die Interviews zeichnen dagegen ein Bild begrenzter Handlungsspielräume, aber auch punktueller Mitwirkung. In einem Fall spricht ein Arzt rückblickend davon, er habe seine „Seele verkauft“ (Söhner et al. 2026). Die institutionelle Doppelrolle als Arzt und Offizier des MfS im Fall des erwähnten Psychiaters erhöhte die Wahrscheinlichkeit von Rollenkonflikten.

Ein abschließender Beweis für den gezielten Einsatz von psychotrop wirkenden Medikamenten zu Verhörzwecken im Stasi-Haftkrankenhaus ist damit noch nicht erbracht. Gleichwohl konnten stichhaltige Indizien aufgezeigt werden, die sich nur aufgrund von „Clausmanns“ Beharrlichkeit während seiner Haft in den Akten niederschlagen konnten. Damit spricht dieser Fall weniger für ein systematisch angelegtes Programm pharmakologischer Vernehmungssteuerung, sondern für einen situativen, opportunistischen Einsatz medizinischer Ressourcen, wenn operative Ziele anderweitig nicht erreichbar schienen. Die entscheidende Erkenntnis liegt in der strukturellen Offenheit des Systems. Das HKH bot dem MfS einen institutionellen Rahmen, in dem medizinische Handlungen – Diagnosen, Medikationen, Gutachten – jederzeit für ein geheimpolizeiliches Kalkül genutzt werden konnten. Dringend anzuregen sind daher weitere Forschungen, die insbesondere die Krankenakten und medizinischen Abläufe im Haftkrankenhaus systematisch untersuchen und mit der schriftlichen Außenkommunikation des MfS abgleichen. Ein solcher Quellenzugriff erscheint vielversprechend, um zu klären, ob, in welchem Umfang und auf welche Weise das Haftkrankenhaus und sein Personal über diesen Einzelfall hinaus in geheimpolizeiliche Praktiken eingebunden waren.

## Danksagung

Für die wertvolle Hilfe bedanken sich die Autorinnen und Autoren insbesondere bei dem für Geschichte der Pharmazie an der Universität Greifswald zuständigen Prof. Dr. Ulrich Meyer. Darüber hinaus bedanken wir uns bei Herrn Dr. Stefan Donth vom Zeitzeugenarchiv der Gedenkstätte Hohenschönhausen für seine Unterstützung bei der Recherche.

## Data Availability

Alle dieser Arbeit zugrunde liegenden Daten sind in diesem Artikel enthalten.

## Footnotes

1 Sofern die Forschungsarbeiten zu einzelnen Untersuchungshaftanstalten inhaltliche Schwerpunkte setzten, betrafen diese mentalitäts- oder geschlechtsgeschichtliche Aspekte; siehe Bastian (2012); Martin (2014).

2 Zu dem vom BMFTR geförderten Forschungsprojekt SiSaP (Seelenarbeit im Sozialismus) siehe Kumbier (2020); Kumbier & Steinberg 2018; Kumbier & Haack 2023 sowie Strauß et al. 2022.

3 Stellenplan des Haftkrankenhauses, undatiert, vermutlich 1987/88, BArch MfS HA Kusch Nr. 28772, Bl. 1–4.

4 Zum Verhältnis der Linie IX zu anderen MfS-Diensteinheiten, insbesondere zur Linie XIV, siehe Engelmann & Joestel (2016: 30).

5 Zum vorrangig rechtshistorisch verstandenen Konzept des Doppelstaates siehe Fraenkel (1984). Zur Anwendung von Fraenkels Konzept auf die DDR siehe Schwan (2009).

6 Einleitungsbericht zur Durchführung einer operativen Personenkontrolle vom 13.12.1985, BArch MfS BV Halle AOP 3255/86, Bl. 8.

7 Eröffnungsbericht zum Anlegen eines Operativ-Vorganges „Inspirator“ der Kreisdienststelle Wittenberg vom 20.03.1986, BArch MfS BV Halle AOP 3255/86, Bd. 1, Bl. 22–25, sowie Eröffnungsbericht zum Anlegen eines Operativ-Vorganges „Inspirator“ vom 20.03.1986, BArch MfS BV Halle 840/87, Bd. 1, Bl. 86–89.

8 Wolfgang Vogel (1925–2008) war ein bekannter Rechtsanwalt in der DDR und später u. a. Unterhändler der DDR beim Freikauf politischer Gefangener durch die Bundesregierung.

9 Strafrechtliche Einschätzung der im Operativen Vorgang „Inspirator“ der Kreisdienststelle Wittenberg erarbeiteten inoffiziellen und offiziellen Beweise und Informationen vom 03.03.1986, BArch MfS BV Halle 840/87, Bd. 1, Bl. 72–84.

10 Einleitungsbericht der örtlichen MfS-Kreisdienststelle zur Operativen Personenkontrolle (OPK), am 29.03.1984, BArch MfS BV Pdm AOP Nr. 1983/95, Bl.31. Zum Sozialprofil von Ausreiseantragstellern siehe Hürtgen, Renate (2014). *Herrschaft im Kreis Halberstadt. Totalitarismus und Demokratie*, 11 (2): 243.

11 Ebd.

12 Operativplan zum Einleitungsbericht der OPK „Joachim“. 02.04.1984, BArch MfS BV Pdm AOP Nr. 1983/85, Bl. 37–38.

13 Ebd.

14 Abschrift eines Briefes von Frau „Müller“ an ihre Tante in West-Berlin vom 19.03.1984, BArch MfS BV Pdm AOP Nr. 1983/85, Bl. 52.

15 Operativplan zum Einleitungsbericht der OPK „Müller“ vom 02.04.1984, BArch MfS BV Pdm AOP Nr. 1983/85, Bl. 38.

16 Aktenvermerk zur Aussprache der örtlichen MfS-Kreisdienststelle mit dem Betriebsdirektor am 18.07.1984, BArch MfS BV Pdm AOP Nr. 1983/85, Bl. 197–198.

17 Ebd.

18 Ebd.

19 Aktennotiz zu einem Vorsprechen von Herrn „Müller“ bei der Abt. Inneres des örtlichen Rates der Stadt am 03.07.1984, BArch MfS BV Pdm AOP Nr. 1983/85, Bl. 193. Auch wenn sich die „Müllers“ in den Quellen auf das Madrider Abkommen berufen, ist davon auszugehen, dass es sich hierbei um die „Verordnung zur Regelung von Fragen der Familienzusammenführung und der Eheschließung zwischen Bürgern der der Deutschen Demokratischen Republik und Ausländern von 1983“ handelt. Vgl. https://www.verfassungen.de/ddr/familienzusammenfuehrung83.htm (15.01.2026).

20 Abschrift einer Eingabe an die Kanzlei des Staatsrates Referat III. am 25.02.1985, BArch MfS BV Pdm Nr. AOP 1983/85, Bl. 440 sowie Bl. 218.

21 Handschriftlicher Vermerk über die erfolgte Hausdurchsuchung am 14.02.1985, BArch MfS BV Pdm AOP Nr. 1983/85 Bl. 359.

22 Abhörprotokoll eines Besuches einer unbekannten Dame bei der Familie „Müller“ am 24.05.1985, streng vertrauliches Schreiben der Abteilung 26 an die örtlichen MfS-Kreisdienststelle, 05.06.1985, BArch MfS BV Pdm AOP Nr. 1983/85 Bl. 459.

23 U‑Plan der Untersuchungsabteilung Potsdam, BArch MfS BV Pdm AOP 1983/85 Bl. 10.

24 Beschluss über das Anlegen eines UV zur Person Herr „Müller“, BArch MfS BV Pdm AU 2642/85, Bd. 1, Bl. 9.

25 BV-Plan der Abt. IX BArch MfS BV Pdm AU 2642/85, Bd. 1, Bl. 19–20.

26 Die Vernichtung der Vernehmungsprotokoll bestätigt ein handschriftlicher Vermerk des MfS, BArch MfS BV Pdm AU Nr. 2642/85, Bd. 1, Bl. 7.

27 Telegramm der BVfS Potsdam, Abt IX. an die HA IX/AKG Auswertung ZKG. Über den Sachverhalt, undatiert, BArch MfS BV Pdm AU 2642/85, Bd. 2, Bl. 12. Ausreiseantragsteller, darunter vor allem Ehepaare mit kleinen Kindern, gingen im Regelfall nicht von einer Inhaftierung aus. Das bestätigen Forschungen von Renate Hürtgen.

28 Verfügung des örtlichen Rates der Stadt zur Heimerziehung des Sohnes der „Müllers“ vom 11.07.1985, BArch MfS BV Pdm AU 2642/85, Bd. 2, Bl. 20.

29 Handschriftliche Vollmacht von Frau „Müller“, datiert auf den 11.07.1985, BArch MfS BV Pdm AU 2642/85, Bd. 2, Bl. 17.

30 Vermerk zur handschriftlichen Vollmacht von Frau „Müller“, datiert auf den 11.07.1985, BArch MfS BV Pdm AU 2642/85, Bd. 2, Bl. 17.

31 Beurteilungsblatt der Abt. IX der BVfS Halle, BArch MfS HA IX Nr. 17564, Bl. 255.

32 Noch am 11.06.1986 wurde in den Akten vermerkt, dass „Clausmann“ jede Aussage und ein Geständnis verweigerte. Antrag des MfS auf Fristverlängerung der U‑Haft bei der Staatsanwaltschaft Halle, BArch MfS BV Halle 840/87, Bd. 1, Bl. 84.

33 Berufungsantrag von Rechtsanwalt Meinzenbach vom 25.08.1986, BArch MfS BV Halle AU 840/87, Bd. 8, Bl. 102–105.

34 Analyseergebnis vom 09.06.1986 des Untersuchungsauftrags an den Operativ-Technischen Sektor (OTS), BArch MfS BV Halle AU 840/87, Bd. 1, Bl. 167.

35 Siehe entsprechende Informationsberichte der Abteilung XIV, BArch MfS BV Halle 840/87, Bd. 1, Bl. 242–244.

36 Verfügung der MfS-BV Halle, Untersuchungsabteilung, BArch MfS BV Halle 840/87, Bd. 6, Bl. 54.

37 Ebd.

38 BArch MfS BV Halle AU 840/87, Bd. 7, Bl. 41–42.

39 Diese Informationen gehen aus einem Brief von „Müller“ an ein befreundetes Paar (unbekannt) vom 05.05.1986 hervor, BArch MfS BV Halle AU 840/87, Bd. 2, Bl. 219.

40 Ebd.

41 Vermerk über die Absprache mit dem Abteilungsleiter im Ermittlungsverfahren gegen „Clausmann“ am 05.06.1986, BArch MfS BV Halle AU 840/87, Bd. 1, Bl. 148.

42 Notizartiger, handschriftlicher Bericht über eine Exploration aus der Verlaufsdokumentation Psychiatrie vom 09.06.1986, Krankenakte von „Clausmann“, BArch MfS AS, Nr. 11249/89, Bl. 18.

43 Ebd.

44 Übersicht über die Maßnahmen im Ermittlungsverfahren gegen „Clausmann“, BArch MfS BV Halle 840/87, Bd. 1, Bl. 117.

45 Vermerk vom 12.06.1986, BArch MfS BV Halle AU 840/87, Bd. 1, Bl. 107.

46 Verlaufskurve in der Krankenakte von „Clausmann“, BArch MfS AS, Nr. 11249/89, Bl. 27.

47 Schreiben zur Einschätzung der Vernehmungsfähigkeit der Abteilung Haftkrankenhaus vom 02.07.1986, BArch MfS AS Nr. 11249/89, Bl. 17.

48 Vermerk zum Vernehmungsprotokoll vom 01.07.1986, BArch MfS BV Halle AU 840/87, Bd. 7, Bl. 57–58.

49 Vernehmungsprotokoll „Clausmanns“ am 01.07.1986, BArch MfS BV Halle AU 840/87, Bd. 7, Bl. 53 sowie Vermerk vom 01.07.1986, BArch MfS BV Halle AU 840/87, Bd. 7, Bl. 58.

50 Wörtlich heißt es darin: „Ich kann dazu nichts sagen […] Filmriß. Ich bringe nichts zusammen. Nach längerem Überlegen“. Vernehmungsprotokoll vom 12.06.1986, BArch MfS BV Halle AU 840/87, Bd. 7, Bl. 41.

51 Vermerk vom 01.07.1986, BArch MfS BV Halle AU 840/87, Bd. 7, Bl. 58.

52 Geheime Absprache des MfS mit der Staatsanwaltschaft Halle zum Ermittlungsverfahren gegen „Clausmann“ am 24.07.1986 in Halle, BArch MfS BV Halle AU 840/87, Bd. 1, Bl. 133.

53 Untersuchungsplan der Abteilung IX der MfS-BV Halle vom Mai 1986, BArch MfS BV Halle 840/87, Bd. 1, Bl. 112.

54 Schriftliche Anweisung der BVfS Halle, Abteilung IX an die Abteilung Haftkrankenhaus am 24.07.1986, BArch MfS, AS, Nr. 11249/89, Bl. 9.

55 Geheime Absprache des MfS mit der Staatsanwaltschaft Halle zum Ermittlungsverfahren gegen „Clausmann“ am 24.07.1986 in Halle, BArch MfS BV Halle AU 840/87, Bd. 1, Bl. 133.

56 Schreiben des ZMD, Abteilung Haftkrankenhaus an den Staatsanwalt des Bezirks Halle zur Einschätzung der Haftfähigkeit „Clausmanns“ am 16.07.1986, BArch MfS AS Nr.11249/89, Bl. 15–16.

57 Informationsschreiben der Hauptabteilung VII an die Hauptabteilung IX vom 07.05.1987, BArch, MfS, HA IX, Nr. 17222, Bl. 84. Zum Freispruchplädoyer von „Clausmanns“ Anwalt siehe Berufungsantrag von Rechtsanwalt Meinzenbach vom 25.08.1986, BArch MfS BV Halle AU 840/87, Bd. 8, Bl. 102–105.
